# Identification of Flap Endonuclease 1 With Diagnostic and Prognostic Value in Breast Cancer

**DOI:** 10.3389/fonc.2021.603114

**Published:** 2021-06-30

**Authors:** Min Wu, Pan Zhang, Penghui Wang, Zhen Fang, Yaqin Zhu

**Affiliations:** ^1^ Institute of Translational Medicine, Medical College, Yangzhou University, Yangzhou, China; ^2^ Jiangsu Key Laboratory of Integrated Traditional Chinese and Western Medicine for Prevention and Treatment of Senile Diseases, Medical College, Yangzhou University, Yangzhou, China; ^3^ Department of Laboratory Medicine, Northern Jiangsu People’s Hospital, Yangzhou, China; ^4^ Department of Medical Laboratory, The Affiliated Hospital of Yangzhou University, Yangzhou, China; ^5^ Department of Cardiology, Northern Jiangsu People’s Hospital, Yangzhou, China

**Keywords:** FEN1, biomarker, breast cancer, diagnosis, prognosis

## Abstract

**Objective:**

This study aims to identify the potential value of flap endonuclease 1 (FEN1) as a diagnostic and prognostic marker for breast cancer (BC).

**Methods:**

ELISA was used to measure serum FEN1 levels and ECLIA for CA153 and CEA levels. Receiver operating characteristic (ROC) curve analysis was used to evaluate the diagnostic value. Oncomine and UALCAN databases were used to analyze the differences in FEN1 mRNA and protein expressions. Kaplan-Meier Plotter database was then used to assess the prognostic value.

**Results:**

Bioinformatics analysis showed that the FEN1 mRNA and protein levels were significantly higher in BC tissues than in normal tissues. FEN1 was detected in culture medium of BC cell lines and serum FEN1 concentrations were significantly increased in BC patients than in cancer-free individuals. Besides, FEN1 exhibited higher diagnostic accuracy (AUC values>0.800) than CA153 and CEA for distinguishing BC patients, especially early BC, from the healthy and benign groups, or individually. Additionally, serum FEN1 levels were significantly associated with the stage (P=0.001) and lymph invasion (P=0.016), and serum FEN1 levels were increased with the development of BC. Furthermore, serum FEN1 levels were significantly decreased in post-operative patients than in pre-operative patients (P=0.016). Based on the Kaplan-Meier Plotter database, the survival analysis indicated that FEN1 overexpression was associated with poor prognoses for overall survival (OS), relapse-free survival (RFS), and distant metastasis-free survival (DMFS) in BC patients.

**Conclusion:**

FEN1 might be a novel diagnostic and prognostic marker for BC.

## Introduction

Breast cancer (BC) is the most widely diagnosed cancer among women and the leading cause of cancer-related deaths (1). Despite the major advances in BC treatment, the prognosis remains poor. The cancer stage during diagnosis determines BC prognosis (2). The survival rate of early BC is significantly higher than that of advanced BC. Therefore, early diagnosis is necessary to improve survival rates and reduce mortality. Current conventional BC diagnostic methods, including gold standard and mammography, do not identify 10-40% of early BC (3, 4). Tumor markers are easily measured, and their clinical values in BC have been investigated (2). Established tumor markers, such as cancer antigen 153 (CA153) and carcinoembryonic antigen (CEA), are the most widely used to diagnose, monitor, and prognosticate BC (2). However, their clinical value remains controversial because of their low specificity and sensitivity, especially in early BC diagnosis (1). Therefore, highly sensitive and specific markers should be discovered to improve early BC detection.

Flap endonuclease 1 (FEN1) is a multifunctional, structure-specific nuclease that contains flap endonuclease (FEN) activity, gap endonuclease (GEN) activity, and exonuclease (EXO) activity (5). These multiple nuclease activities allow FEN1 to participate in numerous DNA metabolic pathways including Okazaki fragment maturation, DNA repair, apoptosis-induced DNA fragmentation, and telomere stability maintenance (6–9). Because of its essential roles, deficiencies in FEN1 function or deletion of the *FEN1* gene would result in predisposition to cancer (10) and rapid tumor development (11). Previously, adenomatous polyposis coli (APC) has been shown to interact with and block FEN1 activity in long-patch base excision repair (LP-BER), thus acting as a susceptibility factor for BC (12). Lin et al. reported that the FEN1 E359K germline mutation abolished FEN1 interaction with Werner Syndrome protein (WRN), an interaction essential for resolving stalled DNA replication forks, and disrupted FEN1 GEN activity, causing aneuploidy-associated cancers in a mouse model (13). Moreover, FEN1 L209P variant expression was prone to induce cellular transformation and tumor growth in a mouse xenograft model (14). Two *FEN1* single nucleotide polymorphisms (69G>A and 4150G>T) were associated with high risk in various cancers (15, 16). However, FEN1 is also upregulated in numerous tumors, including BC, non-small-cell lung cancer (NSCLC), and ovarian cancer (17–21). Knockdown of FEN1 resulted in cell cycle arrest and suppressed cellular proliferation in NSCLC cells (19). Furthermore, SC13, a FEN1 inhibitor, showed cytotoxic and inhibitory activity in human breast cancer in a mouse model (22). Wang et al. reported that FEN1 overexpression in gastric cancer was linked to tumor size, lymphatic metastasis, and differentiation degree (23). Moreover, FEN1 overexpression in ovarian epithelial cancer was correlated with a high cancer grade and stage, and poorer survival (20).

Therefore, FEN1 can serve as both a novel therapeutic target and a promising biomarker. Substantial research has been undertaken regarding the role of FEN1 in the onset and progression of different cancers. However, a systematic analysis of its potential value as a diagnostic and prognostic marker, particularly the possibility of utilizing serum FEN1 as a biomarker, is still unavailable.

In this study, FEN1 was present in the culture medium of BC cells and the serum of BC patients. Besides, serum FEN1 levels were higher in BC patients than in the control groups. Herein, a comprehensive analysis of the diagnostic and prognostic potential of FEN1, including mRNA expression level, tissue protein level, and serum protein level was investigated. At serum level, the efficacy of serum FEN1 in the diagnosis and prognosis of BC and the correlation between serum FEN1 levels and clinicopathological features were investigated. Additionally, the results were compared with known tumor markers (CA153 and CEA) to determine the potential significance of FEN1.

## Materials and Methods

### Study Population

Participants were consecutively enrolled in this prospective observational study in the Northern Jiangsu People’s Hospital between March 2019 and December 2019. Inclusion criteria were: (a) patients pathologically diagnosed with BC (BC group), (b) patients pathologically diagnosed with benign breast diseases (breast hyperplasia, breast cysts, etc.) (benign group), (c) women with normal physical and mammography examinations results (healthy group), and (d) serum tumor markers detected within two weeks before surgery. Exclusion criteria were: (a) male patients; (b) incomplete medical record; (c) history of other primary or secondary tumors; (d) neoadjuvant radiotherapy, chemotherapy, or endocrinotherapy.

A total of 51, 30, and 28 participants were included in the BC, benign, and healthy groups, respectively. Paired post-operative blood samples were obtained from 20 BC patients. The median age in the BC, benign and healthy groups was 44 years (20‐65 years), 44 years (21‐72 years), and 45 years (23‐70 years), respectively.

The Ethics Committee of Northern Jiangsu People’s Hospital approved this study. Informed consent was obtained from all participants following the relevant regulations.

### Protein Concentration in Cell Culture Medium

BC cell lines (MCF-7 and MDA-MB-231) and normal breast epithelial cell line (MCF-10A) were first cultured in Dulbecco’s Modified Eagle Medium (DMEM) for 24 hours, then transferred to FBS-free DMEM for 12 hours. The culture medium was then transferred into an EP tube and centrifuged at 2,000 ×g for 5 minutes. The supernatant was then transferred into an Amicon^R^ Ultra centrifugal filter (Merck Millipore, St. Louis, MO)following a previous report (24), and then centrifuged at 14,000 ×g for 30 minutes. The filter was then placed upside down on a clean microcentrifuge tube and centrifuged at 1,000 ×g for 2 minutes to transfer the concentrated sample to the tube.

### Western Blotting

Protein samples were subjected to sodium dodecyl sulfate-polyacrylamide gel electrophoresis (SDS-PAGE) and then transferred onto polyvinylidene difluoride (PVDF) membranes. The 5% (w/v) skim milk in tris-buffered saline (TBS) and Polysorbate 20 (TBS-T) was used to block the membranes at room temperature for 1 hour. The sample was then incubated with the primary anti-FEN1 antibody (Abcam, Cambridge, UK) at 4°C for 12 hours, then with the corresponding secondary antibody at room temperature for 1 hour. The sample was washed thrice using TBS-T after each incubation. ECL™ Western Blotting Detection reagents were used to visualize the bands.

### Detection of Serum Samples and Pathological Features

Serum was isolated from the samples and stored at -80°C for use after centrifugation at 3000 rpm for 10 minutes. FEN1 ELISA Kit (USCN Business Co., Ltd., Wuhan, China) was used following the manufacturer’s instructions to detect the FEN1 concentrations. ECLIA using the automatic chemiluminescence immunoassay system ROCHE E601 (Roche, Basel, Switzerland) was applied to detect CA153 and CEA levels in serum samples. In this study, the normal cut-off values for the markers used were < 35 U/mL for CA153, and < 4.9 ng/mL for CEA, as recommended by the manufacturer.

The immunohistochemistry (IHC) method was used to detect ER, PR, Her2, and Ki-67 expression status. The ER, PR, Her2, and Ki-67 status was considered as positivity or negativity in accordance with previous study (25). Briefly, ER-positive and PR-positive were defined as the presence of 1% nuclear-stained cells. Her2-positive was indicated by a 3+ score from the IHC evaluation. The Ki-67 staining was considered to be positive if the percentage was >14%. Breast cancer intrinsic subtypes were classified into luminal A, luminal B, Her2-enriched, and triple-negative (26).

### Oncomine Database Analysis

The Oncomine database is an online microarray database with 715 gene expression datasets from 86,733 cancerous and normal samples (27). The Oncomine database was used to determine the differences in FEN1 mRNA expression between tumors and normal tissues in breast cancer type. The detailed steps were as follows: Gene→FEN1; Primary Filters→Differential Analysis→Cancer *vs.* Normal Analysis→Breast Cancer *vs.* Normal Analysis; Dataset Filters→Data Type→mRNA; Datasets→P VALUE= 0.01, FOLD CHANGE = 2, GENE RANK = 10%.

### UALCAN Database Analysis

UALCAN is an interactive web resource for analyzing cancer data and provides protein expression analysis using data from the Clinical Proteomic Tumor Analysis Consortium (CPTAC) Confirmatory/Discovery dataset for BC (28). In this study, UALCAN was used to determine FEN1 proteomic expression profiles based on sample types and individual cancer stages. The detailed steps were as follows: CPTAC analysis→Gene→FEN1→CPTAC dataset→Breast cancer→Total-Protein→Sample types/Individual cancer stages.

### Kaplan-Meier Plotter Database Analysis

The prognostic significance of FEN1 mRNA expression in BC was evaluated using the Kaplan-Meier plotter (www.kmplot.com), an online database capable of assessing the effects of 54,675 genes on survival using 10,461 cancer samples (29). To evaluate the prognostic value of FEN1, samples were divided into two cohorts according to the median expression of FEN1 (high *vs.* low expression). Kaplan-Meier survival plots were then used to analyze the overall survival (OS), relapse-free survival (RFS), and distant metastasis-free survival (DMFS) of BC patients. The detailed steps were as follows: mRNA→Start KM Plotter for breast cancer→Gene symbol→FEN1→Survival→OS/RFS/DMFS→Draw Kaplan-Meier plot.

### Statistical Analysis

Median values with interquartile ranges were used to describe serum protein concentrations. The Mann-Whitney test or paired t-test was used to compare two groups and the Kruskal-Wallis test to compare more than two groups. Receiver operating characteristic (ROC) curve analysis was used to evaluate the diagnostic accuracy and Pearson’s correlation coefficient test to determine the correlation between CA153, CEA, and FEN1 levels in serum. P-value < 0.05 was considered statistically significant. Graph Pad Prism 8.0 (GraphPad, Inc., La Jolla, CA) and SPSS19.0 (IBM Corp., Armonk, NY) were used for all statistical analyses.

## Results

### Identification of FEN1 as a Potential Biomarker Through Bioinformatics Analysis

**Figure 1:**
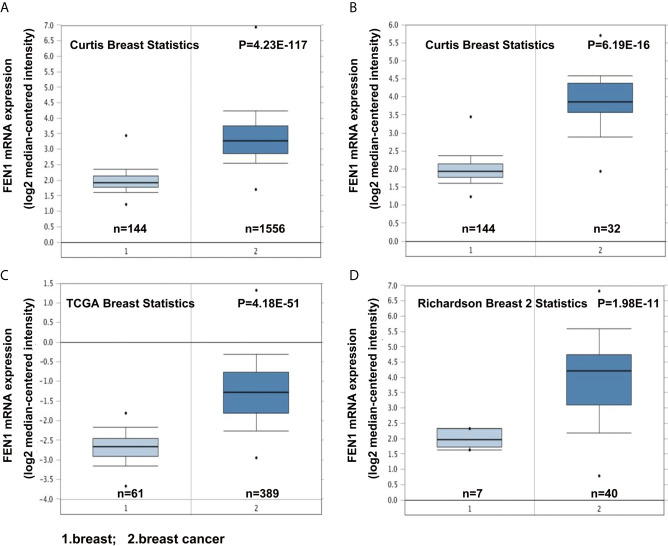
Oncomine database analysis revealing upregulated FEN1 mRNA expression in BC compared with the normal controls. **(A–D)** The FEN1 mRNA levels were significantly higher in BC than in controls according to the Curtis Breast Statistics, TCGA Breast Statistics, and Richardson Breast 2 Statistics datasets.

**Figure 2:**
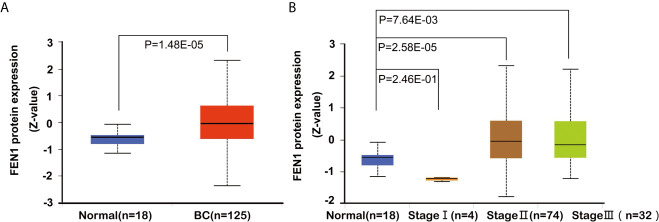
Box plots showing FEN1 expression levels in BC *via* the UALCAN database analysis. **(A)** Box plots show FEN1 protein expression levels in normal tissues *vs.* BC tissues, and **(B)** in normal tissues *vs.* BC tissues in different stages.

The Oncomine database was used to analyze differences in FEN1 mRNA expression between tumor and normal tissues. BC datasets revealed that FEN1 mRNA levels were significantly elevated in BC tissues than normal breast tissues (**Figure 1**). Consistent with the results, using the UALCAN database, FEN1 protein expression levels illustrated significant differences between normal and BC tissues (**Figure 2A**). Moreover, there were substantial differences between normal and BC tissues at different stages (**Figure 2B**).

### The Diagnostic Potential of Serum FEN1 for BC

**Figure 3:**
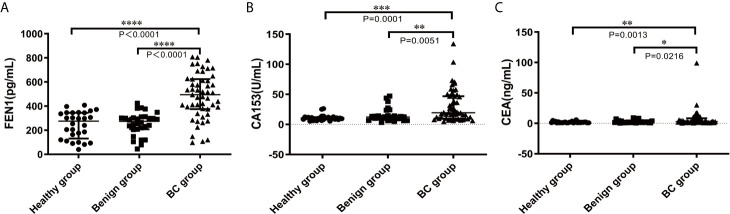
Comparisons of serum FEN1, CA153, and CEA levels in the healthy, benign, and BC groups. **(A)** FEN1. **(B)** CA153. **(C)** CEA. ****P < 0.0001, ***P < 0.001, **P < 0.01, *P < 0.05.

Based on the previous research in our lab, western blot performed revealed that FEN1 was detected in medium of BC cell lines (MCF-7 and MDA-MB-231), but not in normal breast epithelial cell lines (MCF-10A) (**Figure S1**). The result confirmed the presence of FEN1 in the medium and the correlation with its expression levels in the cells. ELISA was used to measure serum FEN1 concentrations with samples in each group. Serum FEN1 levels were significantly higher in BC patients than in patients with benign breast diseases and healthy individuals (**Figure 3A**). FEN1 performance was compared with common BC markers (CA153 and CEA) to assess its diagnostic and prognostic potential in BC. As shown in **Figures 3B, C**, the median levels of CA153 and CEA were significantly increased in the BC group compared with the healthy and benign groups. Furthermore, the three serum proteins showed no significant difference between benign and healthy groups.

**Figure 4:**
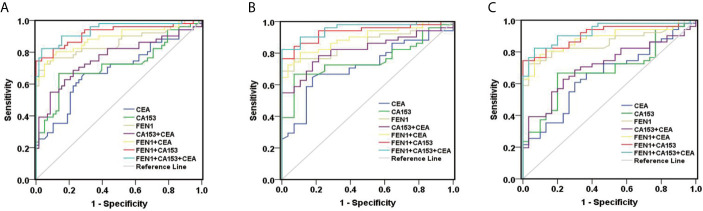
ROC curves constructed to evaluate the diagnostic accuracy of FEN1 for BC. **(A)** For distinguishing BC patients from non-BC subjects. **(B)** For distinguishing BC patients from patients with benign breast diseases. **(C)** For distinguishing BC patients from healthy volunteers.

Receiver operating characteristic (ROC) analysis was performed to determine the diagnostic accuracy of serum FEN1. The area under curve (AUC) value was calculated to evaluate the diagnostic efficacy by constructing ROC curve: low (0.5 ≤ AUC ≤ 0.7), moderate (0.7 ≤ AUC ≤ 0.9) or high (0.9 ≤ AUC ≤ 1) accuracy (30). The curve analysis revealed that FEN1 had a high AUC value (0.860) to distinguish BC patients from non-cancerous individuals. When the cut-off value for serum FEN1 was 389.05 pg/mL, the sensitivity and the specificity were 72.50% and 94.80%, respectively. In contrast, CEA or CA153 all exhibited low sensitivity (66.70%), specificity (70.70%, 86.20%, respectively) and AUC values (0.684, 0.719, respectively). The above indicated that FEN1 was significantly superior to CA153 and CEA in distinguishing BC from the healthy and benign groups. When combined with each other, diagnostic performance had been obviously improved. Among these combinations, FEN1+CEA showed a high sensitivity of 80.40% and FEN1+CA153 had the highest specificity of 100%. Most impressively, the combination of serum FEN1 with CA153 and CEA resulted in a considerable increase in AUC (0.940) with high sensitivity and specificity of 82.40% and 96.60%, respectively (**Figure 4A**, **Table 1**). Similarly, FEN1 also had the best diagnostic potency in distinguishing BC from the healthy (**Figure 4B**, **Table S1**) or benign groups (**Figure 4C**, **Table S2**).

**Table 1:** The diagnostic performances of FEN1, CA153, and CEA in distinguishing BC from the healthy and benign groups.

Index	Sensitivity (%)	Specificity (%)	Youden Index	AUC (95% CI)
CEA	66.70	70.70	0.374	0.684 (0.581,0.786)
CA153	66.70	86.20	0.529	0.719 (0.618,0.820)
FEN1	72.50	94.80	0.674	0.860 (0.785,0.936)
CA153+CEA	68.60	77.60	0.462	0.764 (0.670,0.858)
FEN1+CEA	80.40	87.90	0.683	0.889 (0.824,0.955)
FEN1+CA153	74.50	100.00	0.745	0.920 (0.864,0.977)
FEN1+CA153+CEA	82.40	96.60	0.789	0.940 (0.895,0.986)

FEN1, flap endonuclease 1; CA153, cancer antigen 153; CEA, carcinoembryonic antigen; BC, breast cancer; AUC, area under curve; CI, confidence interval.

**Figure 5:**
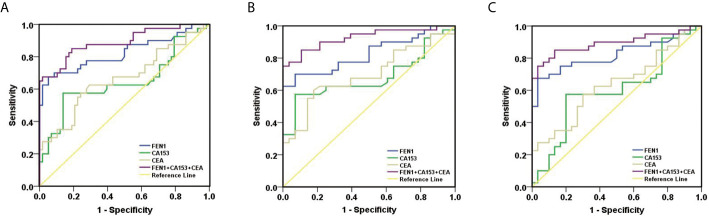
ROC curves constructed to evaluate the diagnostic accuracy of FEN1 for early BC. **(A)** For distinguishing stage I + II BC patients from non-BC subjects. **(B)** For distinguishing stage I + II BC patients from patients with benign breast diseases. **(C)** For distinguishing stage I + II BC patients from healthy volunteers.

To estimate the potential of serum FEN1 in distinguishing early BC (stage I + II) patients from the non-cancerous individuals, separate ROC analysis was performed. FEN1 had the highest AUC value (0.825) with a sensitivity of 67.50% and specificity of 94.80%. CEA and CA153 exhibited lower sensitivity with 57.50% and the specificity of 75.90% and 86.20%, respectively, thus low AUC values of less than 70% (**Figure 5A**, **Table S3**). Analogously, FEN1 showed optimal diagnostic efficacy in differentiating early BC and healthy (**Figure 5B**, **Table S4**) or benign groups (**Figure 5C**, **Table S5**). Notably, when distinguishing stage I + II BC from benign group, CEA and C153 presented low AUC values (0.633, 0.599, respectively), the corresponding P value was greater than 0.05, suggesting no diagnostic value. There was no doubt that the combination of FEN1, CEA, and CA153 exhibited the best diagnostic potency in distinguishing stage I + II BC from non-BC or benign or healthy groups. In addition, their diagnostic parameters positive predictive value (PPV), negative predictive value (NPV) and accuracy for detecting BC were compared. As shown in **Table 2**, these parameters for single measurement in the diagnosis of BC, especially early stage BC, showed the highest values for serum FEN1. Also, the combined measurement in BC diagnosis gave the highest values for all parameters. Interestingly, the combined measurement in early BC diagnosis increased the NPV by 7.8%, but decreased the PPV by 14.66% and the accuracy by 1.04% compared to FEN1 detecting. The above indicated that there was significant value for FEN1 in the diagnosis of early stage of BC.

**Table 2:** Positive predictive values, negative predictive values and accuracies for the detection of BC using markers (%).

Parameters		CEA	CA153	FEN1	FEN1+CA153+CEA
BC *vs.* non-BC	PPV	66.67	80.95	92.50	95.45
	NPV	70.69	74.63	79.71	86.15
	Accuracy	68.81	77.06	84.40	89.91
Early stage BC *vs.* non-BC	PPV	61.11	73.33	89.66	75.00
	NPV	72.13	74.63	80.88	88.68
	Accuracy	68.04	74.22	83.51	82.47

FEN1, flap endonuclease 1; CA153, cancer antigen 153; CEA, carcinoembryonic antigen; BC, breast cancer; PPV, positive predictive value; NPV, negative predictive value.

### The Relationship Between Serum FEN1 Level and the Clinicopathological Characteristics of BC Patients

The relationship between serum FEN1 levels and the clinicopathological features of BC patients was assessed, as shown in **Table 3**. FEN1 levels showed no significant difference in patients’ age distribution (P=0.213). The connection between FEN1 and tumor burden indicators, including tumor size, node status, and stage (31, 32) was estimated. Although serum FEN1 level was increased in patients with large tumor size (>2 cm), there was no relationship between FEN1 levels and tumor size (P=0.513). Remarkably, a positive correlation was noted in the levels of FEN1 among patients at different stages, and the highest FEN1 level was at stage III (P=0.001). Furthermore, the higher FEN1 level presented in patients who were positive for lymph invasion, this difference, compared to the negative group, was also statistically significant (P=0.016). The significantly upregulated FEN1 indicated its role in prognosis prediction in BC patients. Besides, serum FEN1 level was not significantly correlated with histological grades, and patients at grade II showed a higher FEN1 level (P=0.203). The correlation of FEN1 with estrogen receptor (ER) or progesterone receptor (PR) or human epidermal growth factor receptor (Her2) or Ki-67 status was also assessed. Despite the higher FEN1 level in the ER positive or Her2 positive or Ki-67 positive patient group than the negative group, the difference was not statistically significant (P=0.501, P=0.729, P=0.100, respectively). Also, despite the slightly higher FEN1 level in the PR negative group than the PR positive group, there was no significant correlation (P=0.707). Furthermore, FEN1 exhibited differences among various molecular subtypes, with the most elevations occurring in the triple-negative tumor (P=0.854). There was no significant difference between median levels regarding FEN1, CEA, CA153, and histological types (all P >0.05). Moreover, CA153 level was associated with the stage (P=0.041), tumor size (P=0.045), and different molecular subtypes (P=0.038), in BC patients. However, there were no significant associations between CA153 level and age, tumor grade, node status, and Her2/ER/PR/Ki-67 status (all P>0.05). Additionally, there was no statistically significant association between CEA level and pathological features in BC patients (all P>0.05).

**Table 3:** The relationship between serum levels of FEN1, CA153, and CEA and BC clinicopathological features.

Parameters	Groups	n	FEN1 (pg/mL)	P	CA153 (U/mL)	P	CEA (ng/mL)	P
			Median (P25,P75)		Median (P25,P75)		Median (P25,P75)	
Age	20-40	10	414.91 (350.45,495.90)	0.213	21.96 (15.08,51.57)	0.232	2.77 (1.40,3.63)	0.245
	40-60	27	533.69 (410.93,649.51)		15.92 (8.03,36.34)		2.35 (1.14,8.36)	
	≥60	14	488.07 (249.91,631.96)		22.92 (17.30,53.41)		3.98 (2.59,9.75)	
Stage	I	17	394.03 (280.17,494.22)	**0.001**	16.76 (8.67,25.55)	**0.041**	2.23 (1.35,4.02)	0.746
	II	23	516.64 (392.11,579.67)		19.46 (9.21,49.65)		2.68 (1.17,9.35)	
	III	11	728.07 (533.69,780.24)		46.75 (17.65,58.02)		3.13 (2.33,5.83)	
Grade	I	20	414.91 (290.51,558.70)	0.203	18.99 (9.51,43.53)	0.214	2.44 (1.15,7.71)	0.302
	II	17	539.25 (411.10,659.24)		15.92 (7.33,40.59)		3.20 (2.51,10.00)	
	III	14	521.06 (302.60,694.10)		22.92 (16.64,57.05)		2.45 (1.40,3.63)	
Lymphatic Invasion	YES	31	533.69 (409.79,712.29)	**0.016**	16.76 (9.21,36.34)	0.136	2.35 (1.24,4.55)	0.125
	No	20	438.42 (265.84,564.78)		24.67 (16.37,55.62)		3.98 (2.42,9.22)	
Tumor Size	≤2cm	18	429.81 (318.46,630.19)	0.513	12.05 (7.77,36.25)	**0.045**	2.44 (1.16,9.74)	0.529
	>2cm	33	505.59 (385.67,620.65)		20.74 (16.08,54.52)		2.88 (1.82,7.05)	
Her2	Positive	23	519.42 (338.18,613.26)	0.729	18.30 (9.34,45.93)	1.000	2.23 (1.21,4.78)	0.215
	Negative	28	455.89 (376.13,643.07)		19.78 (10.25,48.98)		3.01 (2.34,8.34)	
ER	Positive	36	486.27 (392.59,626.97)	0.501	16.77 (9.24,43.53)	0.123	2.44 (1.22,5.52)	0.109
	Negative	15	338.18 (283.80,613.26)		25.09 (17.65,52.31)		3.20 (2.68,9.50)	
PR	Positive	32	475.09 (382.45,644.14)	0.707	16.53 (9.24,36.31)	0.062	2.77 (1.54,5.57)	0.996
	Negative	19	510.45 (308.87,610.23)		31.51 (17.65,52.31)		2.68 (1.17,9.35)	
Ki-67	Positive	22	536.47 (403.01,699.85)	0.100	32.86 (15.26,49.78)	0.072	2.61 (1.37,9.74)	0.854
	Negative	29	439.60 (323.53,577.15)		16.76 (9.26,36.40)		2.86 (1.63,5.31)	
Subtype	Luminal A	21	420.02 (280.17,611.75)	0.854	16.23 (9.33,22.49)	**0.038**	2.33 (1.35,6.51)	0.148
	Luminal B	10	447.94 (252.83,669.15)		35.22 (11.11,49.78)		1.88 (0.93,8.15)	
	Her2-enriched	12	516.90 (411.08,611.77)		22.59 (9.94,61.22)		2.72 (1.06,7.71)	
	Triple-negative	3	628.04 (472.18,804.99)		58.75 (56.72,103.60)		17.42 (4.78,99.10)	
	Uncertain	5						
Histological type	IDC	35	510.45 (332.43,623.75)	0.764	18.30 (9.68,49.65)	0.382	2.88 (1.80,8.26)	0.729
	ILC	9	494.54 (415.59,601.93)		20.74 (17.62,52.86)		2.23 (1.35,5.63)	
	Others	7	439.60 (262.27,649.51)		16.23 (7.82,25.09)		2.68 (0.90,10.91)	

Bold font represents P<0.05. FEN1, flap endonuclease 1; CA153, cancer antigen 153; CEA, carcinoembryonic antigen; BC, breast cancer; IDC, invasive ductal carcinoma; ILC, invasive lobular carcinoma.

### The Prognostic Potential of FEN1 in BC Patients

**Figure 6:**
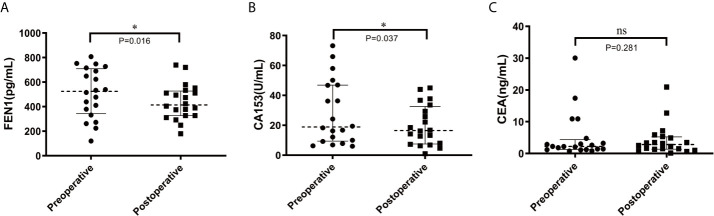
Scatter diagram analysis of the serum levels of FEN1, CA153, and CEA in pre-and post-operative BC patients. **(A)** FEN1. **(B)** CA153. **(C)** CEA. n = 20. *P < 0.05; ns, not significant.

To further determine the prognostic potential of FEN1, serum FEN1 levels in pre-operative and post-operative BC patients were compared. The median FEN1 and CA153 levels were significantly higher in pre-operative patients than in post-operative patients (P=0.016, P=0.037, respectively) (**Figures 6A, B**). However, the median CEA level showed no significant difference between the two groups (P=0.281) (**Figure 6C**).

**Figure 7:**
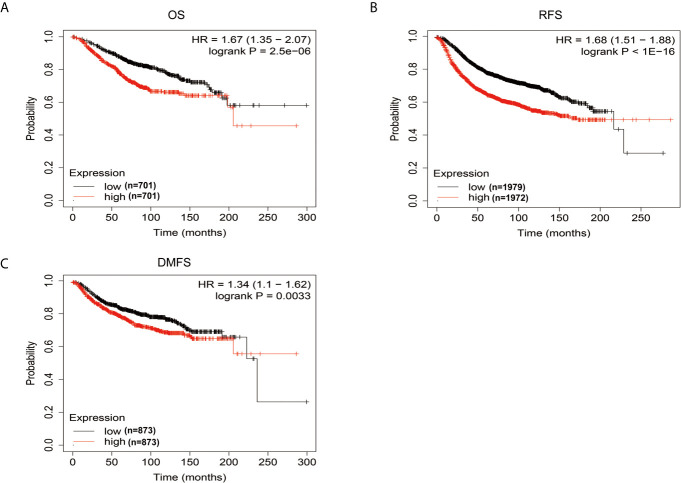
The prognostic information of FEN1 *via* Kaplan-Meier Plotter database. **(A–C)** High FEN1 expression was correlated with poor prognosis regarding OS, RFS, and DMFS in BC patients.

Kaplan-Meier Plotter database was then used to analyze whether there is an association between FEN1 levels and the prognosis of BC patients. The survival analysis indicated that high FEN1 levels were associated with poor prognosis in BC, including OS, RFS, and DMFS (**Figure 7**), revealing a promising prognostic value of FEN1 for BC.

### The Correlation Between CA153, CEA, and FEN1 Levels in Serum

The Pearson’s correlation coefficient was used to analyze the correlation between CA153, CEA, and FEN1 levels in serum. CA153 and CEA levels were not related to the FEN1 levels in the serum (**Table 4**), indicating that FEN1 is an independent marker.

**Table 4:** Pearson association analysis of serum levels of FEN1, CA153, and CEA.

Marker	Pearson’s coefficient	P
CA153	0.164	0.088
CEA	0.146	0.131

FEN1, flap endonuclease 1; CA153, cancer antigen 153; CEA, carcinoembryonic antigen. P < 0.05 is considered as statistically significant.

## Discussion

The use of tumor markers to diagnose BC can effectively improve sensitivity and specificity while aiding early diagnosis (33). FEN1 participates in various DNA metabolism pathways and contributes to cancer progression and drug resistance. However, its specific mechanism in BC is unknown. Estrogen receptor α (ERα) is a key transcriptional regulator in most breast cancers. Flach’s group demonstrated that FEN1 impacted the transcriptional activity of ERα by facilitating coactivator recruitment to the ERα transcriptional complex. FEN1 blockade in BC induced proteasome-mediated degradation of activated ERα, resulting in loss of ERα-driven gene expression and eradicated tumor cell proliferation (21). Zeng et al. reported that FEN1 mediated miR-200a methylation and promoted breast cancer cell growth *via* MET and EGFR signaling (34). Xu et al. reported that FEN1 promoted the migration and invasion of triple−negative breast cancer (TNBC) cells by modulating the expression level of polo−like kinase 4 (PLK4) (35). These results indicate that FEN1 is an important regulator of BC progression and a potential target for BC treatment. Current studies suggest that FEN1, and not serum FEN1, is involved in BC development. In this study, bioinformatics analysis showed that FEN1 mRNA and protein levels were significantly increased in BC tissues than in normal breast tissues, thereby identifying FEN1 as a potential biomarker. Unexpectedly, UALCAN database showed that FEN1 levels were significantly lower in stage I BC than in normal and stage II BC. Zhao et al. reported that FEN1 expression was higher in stage I gastric cancer tissues (n=56) than in normal tissues (36). The ATCG database showed no significant difference in FEN1 expression between stage I and stage II BC tissues (22). The difference could arise due to: 1)small sample size in stage I BC (n= 4), 2) different databases. This study aimed to evaluate and compare the diagnostic and prognostic value of FEN1 with CA153 and CEA as familiar biomarkers in BC to provide further evidence for the serum-based applicability of the proposed markers (37).

This study demonstrated that FEN1 levels were significantly higher in the supernatant of BC cell lines than in normal breast epithelial cell lines. Importantly, the serum concentration of FEN1 was significantly elevated in BC patients compared with the non-cancerous individuals. Besides, the ROC curve analysis revealed that FEN1 had excellent diagnostic potential (AUC>0.800) in distinguishing BC as well as stage I + II BC patients from the healthy and benign groups or individually. Consistent with previous studies (31, 38, 39), CEA and CA153 levels were elevated in BC patients. However, the levels of FEN1, CA153, and CEA in healthy and benign group were similar with no stastical significance, suggesting that FEN1, CA153, and CEA might not be of potential value in the differential diagnoses of healthy and benign group. In comparison to FEN1, CEA and CA153 exhibited much lower diagnostic efficacy with low AUC values, sensitivity and specificity, but CA153 was still superior to CEA in BC diagnosis, similar to previous reports (40, 41). Particularly, CEA and C153 showed no diagnostic value when distinguishing stage I + II BC patients from the benign group (P>0.05), possible due to their limited sensitivity and specificity during the early stages of disease (42–45). Considered on the low sensitivity and specificity of single marker, combinations of tumor markers have been used to improve diagnostic accuracy in the clinical (33, 46). In this study, we demonstrated considerable improvements in BC diagnosis when FEN1 was combined with CA153 and CEA, resulting in the highest AUC value and sensitivity while maintaining a high level of specificity. Diagnostic parameters (PPV, NPV, accuracy) were also calculated for single or combined measurements using FEN1, CA153 and CEA. These parameters for single measurement in the diagnosis of BC, especially early stage BC, showed the highest values for serum FEN1. Notably, the diagnostic parameters for the combined measurement were not all higher than those for the single measurement of FEN1 in detecting early stage BC. The above indicated that FEN1 can function as a novel diagnostic biomarker for BC, especially in early BC.

FEN1 expression had a positive correlation with differentiation degree, lymphatic metastasis, tumor size, and gastric cancer stage (23). Moreover, FEN1 overexpression was correlated with a high grade and stage of ovarian epithelial cancer (20). This research demonstrated that serum FEN1 levels were significantly associated with stage and lymph node status. Moreover, serum FEN1 levels were increased with BC development, suggesting its prognostic value to monitor BC progression. Some clinicopathological factors such as ER and PR are tumor markers that can effectively predict hormonal responsiveness. Her2 is used to estimate prognosis (2), and Ki-67 as a proliferation index. Their expression assessment guides the first-line treatment with respect to targeted approaches (47). Tarek M.A. et al. reported that FEN1 mRNA overexpression was significantly associated with ER negative and PR negative (20). In this study, although there was no significant difference between serum FEN1 level and ER/PR/Her2/Ki-67 status, its level was elevated in patients with PR negative or ER/Her2/Ki-67 positive. Additionally, molecular subtypes based on the results of ER, PR, Her-2, and Ki-67 are of great importance for clinicians. Unfortunately, despite the most elevations occurring in the triple-negative tumor, this study did not find any statistical differences of serum FEN1 among the four subtypes. The serum FEN1 levels of patients before and after surgery were compared to further determine the prognostic potential of FEN1. Notably, FEN1 levels were significantly lower in post- than pre-operative BC patients. Furthermore, the survival analysis *via* the Kaplan-Meier Plotter database indicated that high FEN1 levels predicted a poor prognosis in BC. Taken together, the data suggest that FEN1 expression has prognostic and predictive significance in BC.

Previous studies have demonstrated that CA153 and CEA were also correlated with key pathological features such as tumor size, node status, and TNM stage [33, 34]. In this study, only CA153 levels were linked to stage, tumor size, and molecular subtypes. There was no association between CEA levels and the pathological features of BC patients. Lian et al. reported that CA153 and CEA did not correlate with ER/PR/Her2 status. However, they stated that CEA levels exhibited statistical differences between PR-negative and PR-positive groups (2). Different from our results, Geng et al. concluded that CEA levels in metastatic breast cancer were associated with breast cancer molecular subtypes (48). Also, Wu et al. reported that CEA levels were the lowest in patients with TNBC, and CA153 did not correlate with molecular subtypes (31). The following can explain this difference: first, only patients at stage I‐III BC were investigated, patients at higher stages were not included and second, although novel findings were obtained, further studies using larger sample sizes are needed. Additionally, Pearson’s coefficient showed that FEN1 was an independent marker and was not correlated with CA153 and CEA.

This paper presents new and systematic evidence for the diagnostic and prognostic significance of FEN1 in BC. However, it also raises some questions, including how FEN1 is secreted into the extracellular space or serum, if serum FEN1 levels are related to intracellular FEN1 levels and whether FEN1 is a tumor-specific marker. Nuclear protein secretion is a complex process that requires a tightly controlled relocation program. High mobility group box chromosomal protein 1 (HMGB1) is a ubiquitous nuclear protein that promotes inflammation when extracellularly released after cellular activation, stress, damage, or death (49). HMGB1 hyperacetylation caused its relocalization to the cytosol and secretion (50). Analogously, FEN1 acetylation *via* histone acetylase p300 significantly reduced FEN1’s DNA binding and nuclease activity (51), which may promote FEN1 translocation and secretion. Additional research are needed to elucidate the interrelated mechanism of FEN1 in the future comprehensively.

In summary, this study found that serum FEN1 levels were significantly elevated in BC patients than in non-cancerous individuals. FEN1 exhibited higher diagnostic accuracy (AUC value>0.800) than CA153 and CEA for distinguishing BC patients, especially in early BC. Additionally, serum FEN1 levels were increased with BC development. Decreased FEN1 level was also observed in post-operative patients, suggesting that it can be used to monitor tumor progression and predict the prognosis of BC patients. Database analysis showed that FEN1 exhibited satisfactory differential diagnosis ability and reliable prognosis prediction, thus a promising clinical application prospect.

## Data Availability Statement

The raw data supporting the conclusions of this article will be made available by the authors, without undue reservation.

## Ethics Statement

The studies involving human participants were reviewed and approved by the Ethics Committee of Northern Jiangsu People’s Hospital. The patients/participants provided their written informed consent to participate in this study.

## Author Contributions

MW and YZ designed the study. MW wrote the manuscript. PZ and ZF analyzed the data and interpreted results. PZ and PW worked on patient serum and clinical data collection. PW and YZ reviewed and revised the manuscript. All authors contributed to the interpretation of the findings and critically commented on the manuscript. All authors contributed to the article and approved the submitted version.

## Funding

This research was supported by the National Natural Science Foundation of China (No. 81902114) and the Science and Technology Innovation Cultivation Foundation of Yangzhou University (No. 2019CXJ177).

## Conflict of Interest

The authors declare that the research was conducted in the absence of any commercial or financial relationships that could be construed as a potential conflict of interest. 
