# Supplementary figures and images for: Identification of Flap Endonuclease 1 With Diagnostic and Prognostic Value in Breast Cancer
Source: Front Oncol. 2021 Jun 30;11:603114. doi: 10.3389/fonc.2021.603114 (PMC8278286; doi:10.3389/fonc.2021.603114)

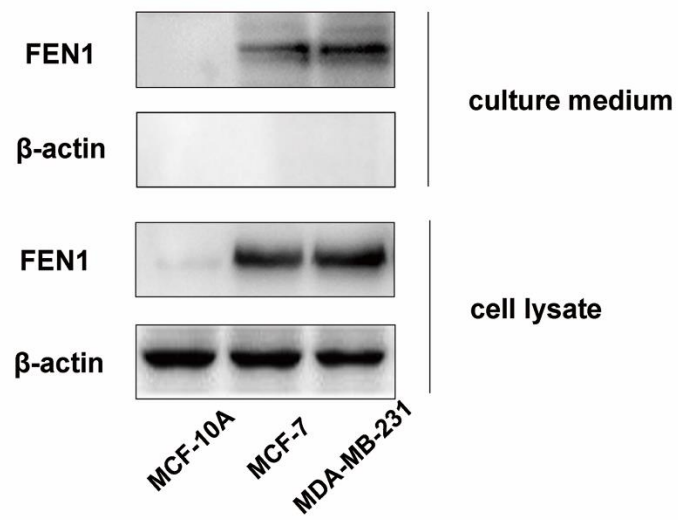

**Figure S1. FEN1 was present in the culture medium of BC cells.**

Supplement: Supplementary file 1 [file Image_1.pdf]
