# Supplementary material for: Identification of Flap Endonuclease 1 With Diagnostic and Prognostic Value in Breast Cancer
Source: Front Oncol. 2021 Jun 30;11:603114. doi: 10.3389/fonc.2021.603114 (PMC8278286; doi:10.3389/fonc.2021.603114)
Supplement: Supplementary file 2 [file Table_1.docx]

**Table S1.** The diagnostic performances of FEN1, CA153, and CEA in discriminating BC from the healthy group.

| Index | Sensitivity  (%) | Specificity  (%) | Youden Index | AUC (95% CI) |
| --- | --- | --- | --- | --- |
| CEA | 64.70 | 82.10 | 0.468 | 0.716(0.603,0.830) |
| CA153 | 66.70 | 92.90 | 0.595 | 0.756(0.650,0.862) |
| FEN1 | 76.50 | 92.90 | 0.693 | 0.863(0.784,0.942) |
| CA153+CEA | 78.40 | 78.60 | 0.570 | 0.821(0.730,0.913) |
| FEN1+CEA | 80.40 | 89.30 | 0.697 | 0.895(0.827,0.963) |
| FEN1+CA153 | 84.30 | 92.90 | 0.772 | 0.933(0.879,0.988) |
| FEN1+CA153+CEA | 82.40 | 100.00 | 0.824 | 0.955(0.914,0.997) |

FEN1, flap endonuclease 1; CA153, cancer antigen 153; CEA, carcinoembryonic antigen; BC, breast cancer ; AUC, area under curve; CI, confidence interval.
