# Supplementary material for: Identification of Flap Endonuclease 1 With Diagnostic and Prognostic Value in Breast Cancer
Source: Front Oncol. 2021 Jun 30;11:603114. doi: 10.3389/fonc.2021.603114 (PMC8278286; doi:10.3389/fonc.2021.603114)
Supplement: Supplementary file 3 [file Table_2.docx]

**Table S2.** The diagnostic performances of FEN1, CA153, and CEA in discriminating BC from the benign group.

| Index | Sensitivity  (%) | Specificity  (%) | Youden Index | AUC (95% CI) |
| --- | --- | --- | --- | --- |
| CEA | 60.80 | 70.00 | 0.308 | 0.653(0.533,0.773) |
| CA153 | 66.70 | 80.00 | 0.467 | 0.686(0.569,0.802) |
| FEN1 | 72.50 | 96.70 | 0.692 | 0.858(0.777,0.939) |
| CA153+CEA | 62.70 | 76.70 | 0.394 | 0.710(0.598,0.822) |
| FEN1+CEA | 78.40 | 90.00 | 0.684 | 0.884(0.812,0.957) |
| FEN1+CA153 | 74.50 | 100.00 | 0.745 | 0.908(0.844,0.972) |
| FEN1+CA153+CEA | 82.40 | 93.30 | 0.757 | 0.926(0.870,0.982) |

FEN1, flap endonuclease 1; CA153, cancer antigen 153; CEA, carcinoembryonic antigen; BC, breast cancer ; AUC, area under curve; CI, confidence interval.
