# Supplementary material for: Identification of Flap Endonuclease 1 With Diagnostic and Prognostic Value in Breast Cancer
Source: Front Oncol. 2021 Jun 30;11:603114. doi: 10.3389/fonc.2021.603114 (PMC8278286; doi:10.3389/fonc.2021.603114)
Supplement: Supplementary file 4 [file Table_3.docx]

**Table S3.** The diagnostic performances of FEN1, CA153, and CEA in distinguishing stage Ⅰ+Ⅱ BC from the healthy and benign groups.

| Index | Sensitivity  (%) | Specificity  (%) | Youden Index | AUC (95% CI) | P  value |
| --- | --- | --- | --- | --- | --- |
| CEA | 57.50 | 75.90 | 0.334 | 0.663(0.549,0.777) | ＜0.001 |
| CA153 | 57.50 | 86.20 | 0.437 | 0.646(0.526,0.765) | 0.015 |
| FEN1 | 67.50 | 94.80 | 0.623 | 0.825(0.732,0.918) | ＜0.001 |
| FEN1+  CA153+CEA | 85.00 | 81.00 | 0.660 | 0.889(0.819,0.959) | ＜0.001 |

FEN1, flap endonuclease 1; CA153, cancer antigen 153; CEA, carcinoembryonic antigen; BC, breast cancer ; AUC, area under curve; CI, confidence interval. P < 0.05 is considered as statistically significant.
