# Supplementary material for: Identification of Flap Endonuclease 1 With Diagnostic and Prognostic Value in Breast Cancer
Source: Front Oncol. 2021 Jun 30;11:603114. doi: 10.3389/fonc.2021.603114 (PMC8278286; doi:10.3389/fonc.2021.603114)
Supplement: Supplementary file 5 [file Table_4.docx]

**Table S4.** The diagnostic performances of FEN1, CA153, and CEA in distinguishing stage Ⅰ+Ⅱ BC from the healthy group.

| Index | Sensitivity  (%) | Specificity  (%) | Youden Index | AUC (95% CI) | P  value |
| --- | --- | --- | --- | --- | --- |
| CEA | 60.00 | 82.10 | 0.421 | 0.695(0.571,0.820) | 0.006 |
| CA153 | 57.50 | 92.90 | 0.504 | 0.681(0.553,0.809) | 0.011 |
| FEN1 | 70.00 | 92.90 | 0.629 | 0.827(0.730,0.924) | ＜0.001 |
| FEN1+  CA153+CEA | 75.00 | 100.00 | 0.750 | 0.926(0.864,0.988) | ＜0.001 |

FEN1, flap endonuclease 1; CA153, cancer antigen 153; CEA, carcinoembryonic antigen; BC, breast cancer ; AUC, area under curve; CI, confidence interval. P < 0.05 is considered as statistically significant.
