# Supplementary material for: Identification of Flap Endonuclease 1 With Diagnostic and Prognostic Value in Breast Cancer
Source: Front Oncol. 2021 Jun 30;11:603114. doi: 10.3389/fonc.2021.603114 (PMC8278286; doi:10.3389/fonc.2021.603114)
Supplement: Supplementary file 6 [file Table_5.docx]

**Table S5.** The diagnostic performances of FEN1, CA153, and CEA in distinguishing stage Ⅰ+Ⅱ BC from the benign group.

| Index | Sensitivity  (%) | Specificity  (%) | Youden Index | AUC (95% CI) | P  value |
| --- | --- | --- | --- | --- | --- |
| CEA | 57.50 | 70.00 | 0.275 | 0.633(0.504,0.763) | 0.058 |
| CA153 | 57.50 | 80.00 | 0.375 | 0.599(0.463,0.736) | 0.158 |
| FEN1 | 67.50 | 96.70 | 0.642 | 0.823(0.723,0.922) | ＜0.001 |
| FEN1+  CA153  +CEA | 85.00 | 86.70 | 0.717 | 0.893(0.814,0.971) | ＜0.001 |

FEN1, flap endonuclease 1; CA153, cancer antigen 153; CEA, carcinoembryonic antigen; BC, breast cancer ; AUC, area under curve; CI, confidence interval. P < 0.05 is considered as statistically significant.
